# Supplementary material for: Enhancing automated strabismus classification with limited data: Data augmentation using StyleGAN2-ADA
Source: PLoS One. 2024 May 24;19(5):e0303355. doi: 10.1371/journal.pone.0303355 (PMC11125505; doi:10.1371/journal.pone.0303355)
Supplement: Attachment — Submitted filename: Response_to_Reviewers.pdf [file pone.0303355.s001.pdf]

Response to Reviewers

- We have indicated all the revisions in the “Revised Manuscript with Track Changes” document in **blue text**. Please refer to these changes for your review.

Journal Requirements

1. **Please ensure that your manuscript meets PLOS ONE's style requirements, including those for file naming.**
    - Response

      We have reviewed and revised the format of our paper to align with the guidelines required by the journal.

        - In response to your guidance, we have updated the symbol associated with "These authors contributed equally to this work" on the first page to align with the journal's guidelines.

2. Thank you for stating the following in the Acknowledgments Section of your manuscript: ~. We note that you have provided funding information that is not currently declared in your Funding Statement. However, funding information should not appear in the Acknowledgments section or other areas of your manuscript. We will only publish funding information present in the Funding Statement section of the online submission form. Please remove any funding-related text from the manuscript and let us know how you would like to update your Funding Statement. Currently, your Funding Statement reads as follows: "NO, The funders had no role in study design, data collection and analysis, decision to publish, or preparation of the manuscript." Please include your amended statements within your cover letter; we will change the online submission form on your behalf.
    - Response

      We have removed the content related to funding from the Acknowledgments section of the paper and have reflected the revisions

to the funding statement in the cover letter.

3. We note that the grant information you provided in the ‘Funding Information’ and ‘Financial Disclosure’ sections do not match. When you resubmit, please ensure that you provide the correct grant numbers for the awards you received for your study in the ‘Funding Information’ section.
    - Response

      We have identified and resolved the mismatch issue for resubmission.

4. We note that you have indicated that there are restrictions to data sharing for this study. PLOS only allows data to be available upon request if there are legal or ethical restrictions on sharing data publicly. Before we proceed with your manuscript, please address the following prompts:
   a) If there are ethical or legal restrictions on sharing a de-identified data set, please explain them in detail (e.g., data contain potentially identifying or sensitive patient information, data are owned by a third-party organization, etc.) and who has imposed them (e.g., a Research Ethics Committee or Institutional Review Board, etc.). Please also provide contact information for a data access committee, ethics committee, or other institutional body to which data requests may be sent.
    - Response

      We have engaged in extensive discussions about opening up our data for the scalability of this research. However, as evident from the generated data samples presented in the paper, the data used in our study includes photographs of direct physical features of patients (such as both eyes). The medical team that collected and processed the data internally confirmed that it was possible to some extent to identify the patient's identity even after cropping the data. This led us to conclude that the data used in the research contains sensitive patient information. For this reason, our research team and the data-providing institution (Yangsan Pusan National University Hospital IRB Center, +82-055-360-4722) have decided to restrict the public disclosure of the data during and after the research period.

5. Please note that PLOS ONE has specific guidelines on **code sharing** for submissions in which author-generated code underpins the findings in the manuscript. In these cases, all author-generated code must be made available without restrictions upon publication of the work.

   Please review your reference list to ensure that it is complete and correct. If you have cited papers that have been retracted, please include the rationale for doing so in the manuscript text, or remove these references and replace them with relevant current references. Any changes to the reference list should be mentioned in the rebuttal letter that accompanies your revised manuscript. If you need to cite a retracted article, indicate the article's retracted status in the References list and also include a citation and full reference for the retraction notice.

   - Response(code sharing)

     We fully understand your requirements regarding code disclosure. Accordingly, we have organized the code used for our research and made it available on GitHub. The link to this repository has been included in the revised submission of our manuscript.

   - Response(reference list check)

     We have reviewed our paper's reference list in accordance with the journal's requirements and have once again confirmed that there are no issues.

Response to Comments from Editor and Reviewers

1. **Authors should draw a graphical abstract of this work.**

   - Response

     We have taken your comment into consideration and have created a Graphical Abstract for our paper. It has been attached as a separate document titled "Graphical Abstract" upon resubmission. We are grateful for your comments, as we believe they (the comments) have helped to enhance the understanding of our readers and have also contributed to the improvement of the quality of our paper.

     Thank you for your valuable feedback.

2. **Explain the novelty of the proposed approach.**

   - Response

     In this paper, we propose a method that leverages generative models to enhance the performance of deep learning-based classification models under extremely limited conditions. While this approach has been utilized in other fields, our research introduces this method for the first time in the area of strabismus classification, marking a novel contribution. Specifically, instead of using generic generative models, we demonstrate the capability to create fake data resembling real images through fine-tuning based on the StyleGAN2-ADA model, which maximizes perceptual realism.

     Beyond the technical novelty, our research adds significant originality by analyzing the performance of the model based on the diagnostic consensus of ophthalmologists (referred to as ODCR in the paper) during the validation phase. This highlights the impact of high-quality data composition on performance in medical data-based research, not just model construction. These details are thoroughly described in the sections "Proposed Method and Contributions" and "Conclusion" of the paper.

     To summarize the novelty of our paper:

     1. We are the first to conduct performance improvement research in the field of strabismus classification using generative models.
     2. We successfully implemented a high-quality generative model to create data that closely resembles real images.
     3. We emphasized the importance of high-quality data through performance analysis based on the diagnostic agreement rate (ODCR) of medical experts.

3. **Proofread the entire manuscript once again.**

   - Response

     We have proofread every section of our paper and addressed the necessary revisions. These adjustments are documented in the marked version of the amended manuscript. The issues we considered when performing proofreading are as follows:

1. Converting sentences from passive voice to active voice
2. Correcting grammatically incorrect sentences
3. Modifying symbols or formats that do not conform to the journal’s guidelines
4. Corrected typos in author’s name and affiliation
5. Revising sentences that could be misunderstood
